# Pharmacotherapy in patients with vasomotor disorders

**DOI:** 10.1016/j.ijcha.2023.101267

**Published:** 2023-09-08

**Authors:** C.K.M. Boerhout, R.G.T. Feenstra, T.P. van de Hoef, J.J. Piek, M.A.M. Beijk

**Affiliations:** a Heart Center, Amsterdam UMC, Amsterdam, the Netherlands; b Department of Cardiology, University Medical Centre Utrecht, Utrecht, the Netherlands

**Keywords:** ANOCA, Coronary function testing, Pharmacotherapy, Vasospams, Abnormal vasodilation

## Abstract

**Background:**

Anginal symptoms in patients with non-obstructive coronary artery disease are frequently related to vasomotor disorders of the coronary circulation. Although frequently overlooked, a distinct diagnosis of different vasomotor disorders can be made by intracoronary function testing. Early detection and treatment seems beneficial, but little evidence is available for the medical treatment of these disorders. Nevertheless, there are several pharmacotherapeutic options available to treat these patients and improve quality of life.

**Methods & findings:**

We performed an extensive yet non-systematic literature search to explore available pharmacotherapeutic strategies for addressing vasomotor disorders in individuals experiencing angina and non-obstructive coronary artery disease. This article presents a comprehensive overview of therapeutic possibilities for patients exhibiting abnormal vasoconstriction (such as spasm) and abnormal vasodilation (like coronary microvascular dysfunction).

**Conclusion:**

Treatment of vasomotor disorders can be very challenging, but a general treatment algorithm based on the existing evidence and the best available current practice is feasible.

## Highlights

• Early detection and treatment of vasomotor disorders seems beneficial.
• Several therapeutic options exist to treat vasomotor disorders.
• Treatment algorithms are provided for different vasomotor disorder.

## 1 Introduction

Angina pectoris due to coronary artery disease (CAD) affects over 126 million people globally and is a direct expression of a supply and demand mismatch of myocardial oxygen which results in myocardial ischemia [1]. Historically, it was thought that the pathological process of CAD was solely ascribable to atherosclerotic plaque accumulation in the coronary arteries which progressively trims myocardial blood supply. Yet, up to 50 % of patients with signs and/or symptoms of CAD or ischemia undergoing invasive coronary angiography (ICA) are found to have non-obstructive coronary arteries disease (NOCAD) [2].

Considered a working diagnosis, anginal symptoms in NOCAD (ANOCA) or even ischemia (INOCA) can be related to a differential diagnosis based on distinct vasomotor disorders of the coronary circulation. Although frequently overlooked, intracoronary function testing (ICFT) during ICA can adequately diagnose either abnormal vasoconstriction (i.e. vasospastic angina, Prinzmetal angina), abnormal vasodilation (i.e. variant angina, coronary microvascular dysfunction) or a combination of both [3]. While little evidence is available for medical treatment of vasomotor disorders, there are distinct pharmacotherapeutic options to treat symptoms and improve quality of life [4]. In addition, based on the pathophysiological substrates of vasomotor disorders, some viable options merit consideration when treating ANOCA patients.

The purpose of this review is to provide a comprehensive and practical review of the potential pharmacotherapeutic options available to treat vasomotor disorders in ANOCA patients.

## 2 Pathophysiology of vasomotor disorders

### 2.1 Abnormal vasoconstriction

Angina due to abnormal vasoconstriction (i.e. vasospastic angina, syndrome X, Prinzmetal Angina) is characterized by spontaneous episodes of angina, usually in rest, due to reversible constriction (i.e. spasm) of a coronary artery either in the epicardial (VSA) or the microvasculature (MSA) domain [5]. In the epicardial domain, spasm can occur diffuse across the coronary vessels or focally. The pathophysiology of abnormal vasoconstriction is not fully elucidated, but it considered as the result of several components: (i) endothelial dysfunction, (ii) hyperreactivity of vascular smooth muscle cells (VSMCs) and (iii) transient vasoconstrictor stimulus acting on the hyperreactive VSMCs [6]. In healthy coronary arteries, the endothelial cells play a crucial role in the regulation of the vascular tone through several vasodilators [7]. Endothelial dysfunction, impairing the release of these potent vasodilators, makes the vascular bed susceptible for vasoconstrictive stimuli, especially in the presence of hyperreactive VSMCs [8].

The diagnosis of abnormal vasoconstriction can be made through the intracoronary infusion of vasoconstriction provocative substances like acetylcholine (Ach) or ergonovine [9], [10]. Normally, Ach induces the release of endothelium-dependent relaxation factors (mainly nitric oxide (NO)). However, Ach also has a direct vasoconstrictor effect on the VSMCs. Normally this effect is attenuated by the vasodilator effect of healthy endothelium. However, in the presence of endothelial dysfunction or hyperreactivity of the VSCM, Ach induces vasoconstriction [11].

### 2.2 Abnormal vasodilatation

Angina due to abnormal vasodilation of the coronary circulation is characterized by the inability of the coronary circulation to increase myocardial blood flow supply in order to match myocardial demand [12]. The regulation of myocardial blood flow is mainly sited within the coronary microcirculation and therefore, abnormal vasodilation is commonly referred to as coronary microvascular dysfunction (CMD). Patients with a decreased abnormal vasodilatory capacity often present with a similar clinical presentation as patients with obstructive CAD (i.e. effort-induced angina and/or dyspnea) [13]. In ANOCA patients, the abnormal vasodilator capacity may result from (i) structural remodeling of the coronary microcirculation leading to fixed reduced microcirculatory conductance, or (ii) functional limitations due to an increased resting flow as a result of an increased myocardial metabolism [14]. In patients with an abnormal vasodilator capacity, the hemodynamic response to a non-endothelium dependent vasodilator, such as adenosine, are a reduced coronary flow reserve (CFR), often accompanied by an increased microvascular resistance (Hyperemic microvascular resistance (HMR) and/or index of microvascular resistance (IMR)) [14].

### 2.3 Endotype distinction

**Fig. 1:**
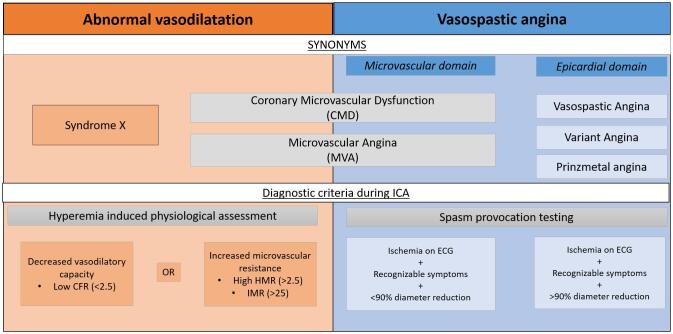
Title: Overview of the synonyms and diagnostic criteria of the distinct vasomotor disorders in patients with angina and non-obstructive coronary artery disease Legend: CMD (coronary microvascular dysfunction); MVA (Microvascular angina); ICA (Invasive coronary angiography); CFR (Coroonary flow reserve); HMR (Hyperaemic Microvascular Resistance); IMR (Index of Microvascular Resistance).

Abnormal vasodilatation and vasoconstriction, either resulting from atherosclerotic process, genetic predisposition or other triggers, may be present by itself or combined in patients with ANOCA [15]. However, considering the different pathological substrates, the distinction provides crucial information to target therapeutic strategies and a comprehensive diagnostic approach is recommended. Fig. 1 depicts an overview of the distinct vasomotor disorders, the synonyms used in current literature and diagnostic criteria to identify them.

### 2.4 Non-pharmacological therapy

Patients with ANOCA due to vasomotor disorders frequently have an increased coronary atherosclerotic burden and endothelial dysfunction [16]. Hence, the first step of treatment should consist of non-pharmacological therapy. Control of traditional risk factors for cardiovascular disease (CVD) is important as it may also contribute to the pathophysiology of vasomotor disorders. Cardiac rehabilitation may also improve clinical conditions in patients diagnosed with vasomotor disorders. Importantly, cessation of smoking is crucial as nicotine is a vasoconstrictor and also induce or exacerbate coronary atherosclerosis [17]. Moreover, taking into account that vasoconstrictive stimuli should be limited, mental stress and/or excessive fatigue must be avoided. Agents that have potential vasoconstriction-inducing properties including antimigraine agents (e.g., triptans) and chemotherapy (e.g., 5-fluorouracil, capecitabine) as well as cocaine, marijuana, amphetamines, alcohol, and ephedrine-based weight-loss products may exacerbate coronary vasoconstriction and should be avoided [17]. Finally, whether specific diets, such as anti-inflammatory, vegan, or Mediterranean, improve symptomatic coronary vascular dysfunction is currently unknown.

### 2.5 Pharmacotherapy

Pharmacological treatment of patients with symptomatic vasomotor disorders is challenging as they represent a heterogeneous group. The distinct vasomotor disorders originate from different pathophysiological substrates that remain elusive in clinical practice and, consequently, benefit from individually specified therapeutic agents [4]. The goal of treatment in patients with ANOCA due to vasomotor disorders, similar to that of obstructive CAD, is either disease-modifying or symptom-reducing. While large, randomized trials on treatment strategies are scarce, there are multiple pharmacotherapeutic options showing positive effects in different ANOCA subgroups. Moreover, based on the theoretical or biological properties, some viable options could be considered.

In this section, we will discuss the different options and their role in the treatment of vasomotor disorders. Table 1 provides a comprehensive overview of the most important evidence with respect to the different pharmacotherapeutic options.

## 3 Disease modifying

### 3.1 Antiplatelet therapy

The role of antiplatelet therapy (APT) is indispensable for the management of stable and acute coronary syndromes associated with atherosclerosis; however, it is less clear in ANOCA patients [18]. Yet, APT is commonly prescribed in patients with ANOCA as a substantial number of these patients have concomitant coronary atherosclerosis [19]. While robust data regarding the use of APT in ANOCA patients is lacking, some data regarding safety and efficacy exist. In patients with abnormal vasoconstriction, high-dose aspirin (>325 mg daily) is known to aggravate coronary vasoconstriction via the inhibition of prostacyclin and should be avoided [20]. Low-dose aspirin (40–100 mg once daily), frequently prescribed as maintenance therapy in patients with anginal symptoms, does not have this vasoconstrictive effect, but does not seem to provide a prognostic benefit in patients with objective abnormal vasoconstriction [20], [21], [22], [23]. In patients with isolated abnormal vasodilatation or mixed vasomotor disorders, the role of ATP has still to be determined. Nevertheless, a substantial percentage of patients with an abnormal vasodilatory capacity have endothelial dysfunction, and although angiography shows no significant plaque burden, IVUS has demonstrated coronary atherosclerosis in most patients [16], [24]. Therefore, several guidelines advocate the use of APT such as aspirin in patients with coronary microvascular dysfunction due to a reduced vasodilator capacity and no obstructive epicardial CAD.

### 3.2 Statin therapy

Similar to ATP therapy, HMG-CoA (3-hydroxy-3-methylglutaryl-coenzyme) reductase inhibitors or ‘statins’ are considered standard of care in patients with anginal symptoms for the prevention of atheromatous plaque formation, progression and complications, with the main goal of reducing cardiac death, acute myocardial infarction, stroke and heart failure [18]. Besides their lipid-lowering effect, statins are beneficial due to their pleiotropic effects on the molecular pathways involved in inflammation and oxidative stress. Statins reduce NADPH-oxidase activity and promote endothelial NO synthase activity directly and via the induction of tetrahydrobiopterin synthesis. As such, statins may help attenuate coronary vasoconstriction [25], [26]. Moreover, statins may improve endothelial function over time and thereby temper the sensitivity of the coronary circulation to vasoconstrictive triggers. The risk-reducing effects of statin therapy in patients with vasomotor disorders have been inconsistent. Most studies evaluated the effect of statin therapy on major adverse cardiac events (MACE) (e.g. myocardial infarction, revascularization or myocardial death) in patients with abnormal vasoconstriction and found no significant risk-reducing effect of statin therapy [27], [28], [29], [30]. Nevertheless, the prognostic benefit of statin therapy in patients with an increased atherosclerotic burden is undisputed [31]. Hence, in patients with an abnormal vasodilator capacity, statins should be considered. Altogether, taking in account the additional pleiotropic and anti-vasoconstrictive effects, statin therapy merits consideration in all patients with vasomotor disorders in proportion to the potential adverse effects such as muscle pain, digestive problems or mental fuzziness.

### 3.3 Renin-angiotensin system inhibitor

Renin–angiotensin system (RAS) inhibitors such as the angiotensin-converting enzyme (ACE) inhibitors and angiotensin receptor blockers (ARBs) may be effective in treating vasomotor disorders since RAS is known to be closely associated with endothelial function, and RAS inhibitors are known to improve endothelial function [32]. The coronary resistance vessels are influenced by multiple factors including ACE and local angiotensin II (AT-II) levels, and CFR measurements in patients with hypertension, diabetes, or CAD suggest direct and long-term improvement of vasodilator capacity with RAS inhibition [33], [34], [35], [36], [37], [38]. These findings support the assumption that blockade of the RAS may regulate microvascular function in response to stressors. Moreover, ACE inhibitors may improve microvascular function by limiting the vasoconstrictor and pro-oxidant effects of AT-II.

The effect of RAS inhibitors in ANOCA patients has been evaluated in multiple studies over the past decades. Either alone or in combination with statin therapy, RAS inhibition seems to improve quality of life and exercise endurance measured during stress tests [39], [40], [41], [42]. The possible mechanism of this effect may be a modulation of coronary tone and the effect of long-term ACE inhibitors on endothelial NO metabolism. In a randomized, placebo-controlled trial with enalapril (5 mg twice daily), ANOCA patients who received Enalapril showed a significant improvement in CFR and exercise duration [41]. Furthermore, compared to placebo, enalapril significantly reduced plasma van Willebrand factor (p = 0.03) and ADMA levels (p = 0.01) and increased NOx levels (p = 0.01) and the ratio of L-arginine to ADMA (p < 0.01). All of which are objective indicators of endothelial function and NO metabolism. The role of RAS in women with ischemia and non-obstructive CAD was also investigated in the WISE study [39]. In a total of 78 patients, ACE inhibition with quinapril significantly improved CFR measurements and was associated with a reduction in angina burden. Interestingly, the beneficial response was limited to women with lower baseline CFR values, suggesting that RAS may be more involved among women with more severely impaired vasodilation. In patients with isolated abnormal vasoconstriction, RAS inhibition seems to lower the incidence of recurrent angina, all-cause mortality, and MACE and some reports exist of successfully treating refractory angina due to multivessel coronary spasm with valsartan on top of maximum antianginal medication [43].

Based on the disease-modifying and risk-reducing effects, RAS inhibitors should be considered as general treatment in patients with objective vasomotor disorders and future research is warranted to further elucidate its potential therapeutic effects. Currently, no reports on the effect of additional neprilysin inhibitor therapy on top of ARB using sacubitril/valsartan are available in the treatment of ANOCA patients and the effect of direct renin inhibitors such as aliskiren in ANOCA patients remains to be determined.

## 4 Symptom reducing

### 4.1 Beta-blocker

Beta-adrenergic receptor blockers (or beta-blockers) are commonly used as first-line therapy to reduce anginal symptoms [18]. Due to the inhibition of the beta-1 receptor-mediated stimulation of heart rate and myocardial contractility, beta-blockers improve the myocardial supply–demand balance. As such, it effectively reduces angina frequency and symptoms in patients with angina due to obstructive CAD or an abnormal vasodilator capacity of the coronary circulation. However, in patients with abnormal vasoconstriction, the use of beta-blockers that do not carry alpha_1_-adrenergic antagonist activity (all beta-adrenergic receptor blockers except labetalol and carvedilol) should be avoided in patients with objective abnormal vasoconstriction as they can exacerbate coronary vasoconstriction [44], [45], [46]. Beta_2_-adrenergic receptor stimulation dilates blood vessels and blockade of beta_2_-adrenergic receptors may lead to unimpeded alpha_1_-adrenergic receptor stimulation, converting the effects of sympathetic stimulation into a more dominant alpha_1_-adrenergic (vasoconstricting) response.

If a beta-blocker is indicated in ANOCA patients, agents with mixed alpha_1_- and beta-adrenergic receptor antagonist properties should be considered, as these agents may have an overall vasodilatation effect. In particular, in patients with proven endothelial dysfunction and impaired vasodilatation beta-blockers are indicated [47].

### 4.2 Calcium-channel blocker

The different subclasses of CCBs include the DHP (amlodipine, nifedipine, barnidipine, lercanidipine) and the non-DHP (diltiazem and verapamil). Both subclasses have similar mechanisms of action in which CCBs induce vasodilation of the peripheral arteries and on the myocardium via inhibition of calcium influx through the L-type calcium channels in excitable membranes [48]. DHP CBBs suppress the influx of calcium intracellularly, resulting in vasodilatation of the peripheral arteries. In contrast, non-DHP CCBs are relatively more negative chronotropic and inotropic, thereby reducing cardiac workload by reducing ventricular contractility and myocardial oxygen demand.

While the use of CCBs in patients with isolated abnormal vasodilatation has still to be elucidated, the safety and efficacy of CCBs in patients with objective abnormal vasoconstriction were studied in several clinical trials and case reports [49], [50], [51], [52], [53], [54], [55], [56]. Studies evaluating either DHP or non-DHP CCBs report a substantial reduction of angina frequency, use of short-acting nitroglycerin and the number of hospitalizations for clinical instability both in the short- and long-term period. One study even reported a significantly lower incidence of MACE in patients with abnormal epicardial vasoconstriction treated with benidipine [53]. These results support that CCBs are highly effective in reducing ischemic episodes in patients with abnormal vasoconstriction and are currently recommended for these patients as first-line agents for the treatment and prevention of anginal symptoms. It is important to note that, due to the extensive first pass metabolism of non-DHP CCBs, higher doses are recommended (e.g. diltiazem up to 200 mg twice daily), an effect not observed with DHPs. Even combined use of DHP and non-DHP CCBs might be required to optimize treatment.

### 4.3 Nitrates

As mentioned before, NO is one of the most important substrates influencing the vasomotor function of the coronary arteries. Both in patients with obstructive CAD and patients with ANOCA, the contribution of NO is disturbed [57]. Several different vasodilator agents that act via the NO-pathway act as NO donors and their anti-anginal effect stems from the ability to reduce myocardial oxygen demand through systemic vasodilation and improvement of myocardial blood flow.

#### 4.3.1 Short- and long-acting nitrates

The use of rapid-acting, sublingual nitro-glycerine is preferred for acute anginal attacks, while long-acting nitrates are important for the chronic treatment of ANOCA as they withhold acute angina attacks and may prevent recurrent attacks [58]. In practice, CCBs are preferred over long-acting nitrates, due to the potential nitrate tolerance probably due to the formation of a superoxide (O2–) molecule [59]. Superoxide causes multiple downstream receptor effects which leads to the inhibition of soluble guanylate cyclase, the enzyme responsible for NO signalling. A nitrate-free interval between the evening dose and morning dose should be warranted. Moreover, there is a biological plausibility that long-term nitrates may have toxic effects in humans and caution is warranted. Currently, long-acting nitrates should be used as second-line agents, when patients with abnormal vasoconstriction are not responding adequately to CCB therapy. In this respect, it is important to note that nitrates seem more effectively in preventing epicardial located vasospasm compared to the prevention of microvascular vasospasm [60]. In patients with an isolated abnormal vasodilator capacity, short-acting nitrates are recommended to relieve spontaneous attacks of angina [61].

#### 4.3.2 Nicorandil

Nicorandil, although structurally a nitrate, differs from classic nitrates in several respects. Nicorandil is a balanced vasodilator affecting both venous and arterial beds. It exerts 2 distinct anti-angina mechanisms, acting as both NO donor and adenosine triphosphate (ATP)-sensitive K+ channel opener [62]. Similar to nitrates, NO acts via cGMP signaling pathways within VSMC causing vasodilatation of the epicardial coronary arteries at low plasma levels [63], [64]. At high plasma concentration, its action on ATP-sensitive sensitive K+ channel results in VSMC hyperpolarization and closure of L-type voltage gated Ca2+ channels which acts to improve vascular endothelial function Moreover, nicorandil dilates coronary microvessels with a diameter of 100–200 mm and thereby reducing coronary arterial resistance, increase blood flow, and achieve the purpose of treating microvascular angina [65].

Data on nicorandil specifically in ANOCA patients is scarce, but a recent *meta*-analysis involving 2323 patients showed the potential of nicorandil for improving angina symptoms, resting ECG, treadmill test result, and endothelial function [66]. An important advantage over long-acting nitrates, is that nicorandil does not cause tolerance or rebound angina. Nicorandil is contraindicated in the setting of hypotension and when the patient is on PDE-5 inhibitors, nicorandil is generally well-tolerated and therefore a viable option for patients with ANOCA.

#### 4.3.3 Molsidomine

Molsidomine is similar to long-acting nitrates, both in terms of mechanism of action and efficacy. It is a prodrug that is hydrolyzed to linsidomine in the liver via first-pass effect, which subsequently releases NO. Linsidomine increases levels of cGMP, decreasing levels of intracellular Ca2+ in smooth muscle cells leading to relaxation of VSMC. It thereby leads to increased myocardial perfusion by vasodilatation of the coronary arteries, and reduced oxygen demand by increasing peripheral venous vasodilation and decreasing cardiac preload and wall tension [67]. Moreover, molsidomine inhibits platelet aggregation and has indirect anti-proliferative effect [68], [69]. Similar to nicorandil, molsidomine does not induce any meaningful tolerance. Randomized trials evaluating the clinical efficacy of molsidomine in ANOCA patients are lacking.

### 4.4 Alpha1-adrenergic receptors antagonist

The alpha_1_-adrenergic receptors are members of the G-protein-coupled receptor (GPCR) superfamily and consists of 3 highly homologous subtypes, including α_1A_-, α_1B_-, and α_1D_-adrenergic. Upon activation, a heterotrimeric G-protein (G_q/11_) activates phospholipase C, which causes phosphatidylinositol to be transformed into inositol triphosphate (IP3) and diacylglycerol. Thereafter, IP3 diffuses into the cytosol and to find the IP3-receptor on the endoplasmic reticulum, triggering calcium release. Subsequently, alpha_1_-receptors primarily mediate smooth muscle contraction [70].

The effects of alpha_1_-adrenergic receptors on abnormal coronary vasoconstriction are not yet elucidated as there is limited and conflicting evidence [70], [71], [72], [73], [74], [75].

### 4.5 Ivabradine

Ivabradine reduces the heart rate through inhibition of the I_f_ channel, the main determinant of the pacemaker function of the sinus node, without influencing the other currents involved in the genesis of action potentials in the sinus node cells [76]. As such, ivabradine decreases the heart rate and myocardial oxygen consumption at rest and during exercise but has no other direct cardiovascular effects (negative inotropic effect, blood pressure reduction) [77]. In patients with stable obstructive CAD, ivabradine possesses well-documented antianginal and anti-ischemic properties comparable to other antianginal agents, such as beta-blockers and CCBs. Moreover, it was shown that ivabradine improved hyperaemic coronary flow velocity and CFR [78]. These effects remained even after heart rate correction indicating improved microvascular function. Ivabradine significantly improved Seattle Angina Questionnaire (SAQ) items and EuroQoL scale compared with placebo. No effects on coronary microvascular dilatation in response to adenosine, cold pressor test or on peripheral endothelial function (by flow-mediated dilation) were observed with ivabradine or placebo [79]. Currently, there are no studies that have evaluated the effect of ivabradine in patients with abnormal vasoconstriction.

### 4.6 Ranolazine

Ranolazine is an anti-ischemic agent that seems to mainly act by improving left ventricular diastolic function through selective inhibition of the late sodium current in cardiomyocytes, which prevents intra-cellular calcium overload during ischemia and decreases oxygen consumption in the cardiomyocytes [80]. Some studies suggest that ranolazine also has anti‐inflammatory or antioxidant effects which may improve glycometabolic homeostasis, which is important in abnormal vasodilatation [81]. Ranolazine does not cause significant haemodynamic changes and may be used concomitantly with other anti-anginal or disease-modifying therapies. However, ranolazine is metabolized by the CYP3A enzyme and inhibits the metabolizing enzyme, cytochrome CYP2D6. Caution and dose modification is needed of ranolazine and concomitant use of other drugs that interact with those enzymes (i.e. diltiazem, verapamil, tricyclic antidepressants).

### 4.7 Cilostazol

Cilostazol is a selective phosphodiesterase-3 inhibitor which produces vasodilation, antiplatelet activity, improved blood flow, and inhibition of vascular smooth muscle cell growth. Cilostazol increases cyclic adenosine monophosphate (cAMP) levels that results in an increase in the active form of protein kinase A (PKA), which is directly related with an inhibition in platelet aggregation. PKA also prevents the activation of myosin light-chain kinase, important in the contraction of smooth muscle cells, thereby exerting its vasodilatory effect [82]. Some studies have indicated that cilostazol treatment increases both CFR and flow-dependent coronary dilation and had an additional angina-reducing effect in combination with a CCB in patients with abnormal vasoconstriction [82], [83]. The long-term safety and efficacy of cilostazol in the treatment of ANOCA is not extensively studied and therefore, cilostazol may be appropriate as add-on therapy in patients who fail conventional therapy with CCBs.

### 4.8 Endothelin receptor antagonists

The human endothelin (ET) family consists of 3 isopeptides: ET-1, ET-2, and ET-3. Only ET-1 plays an important physiological and pathophysiological role, especially in the regulation of the vascular tone [84]. ET-1 is predominantly released from vascular endothelial cells and its paracrine effect induces an extremely potent and long-lasting vasoconstrictor action. Moreover, ET-1 stimulates the generation of other local mediators of the vascular tone, including NO, prostacyclin, and platelet-activating factor [85]. Importantly, ET-1 receptors have 2 subtypes, ET_A_ and ET_B_. Stimulation of ET_A_ receptors in smooth muscle cells results in vasoconstriction, whereas, stimulation of ET_B_ receptors that are present in endothelial and vascular smooth muscle cells has both vasoconstrictive and dilatory effects [86]. It is becoming increasingly clear that an imbalance between ET-1 and NO is a characteristic of endothelial dysfunction and increased levels of ET-1 have been linked to coronary vasospasm [87]. In addition, ET-1 levels are associated with impaired coronary vasodilatory response. There are 3 main endothelin receptor antagonists (ERA): selective ET_A_ receptor antagonists (i.e. ambrisentan), dual ET_A_/ET_B_ antagonists (i.e. bosentan, macitentan), and selective ET_B_ receptor antagonists.

The evidence of ERA therapy among CAD (both obstructive and non-obstructive) is limited. Intracoronary administration of bosentan was shown to induce coronary vasodilatation and two cases reports have documented beneficial effects of bosentan in the treatment of abnormal vasoconstriction [88], [89]. Currently, 2 studies are ongoing evaluating ERA. The results of the double-blind, placebo-controlled, crossover VERA (Vasospastic angina treatment by Endothelin Receptor Antagonism) trial are expected this year (www.clinicaltrialsregister.eu, EudraCT Number: 2018–002623-42). The multicentre European, placebo-controlled, crossover design “Precision Medicine With Zibotentan in Microvascular Angina (PRIZE)” study is enrolling and will evaluate 356 patients with abnormal vasodilatory capacity in terms of exercise duration without angina.

Based on the limited available evidence and the severity of adverse effects, use of bosentan (or other ERAs) to treat vasomotor disorders should be installed with caution and may be used as a last option to control angina that is that is refractory to all other conventional medical therapies.

### 4.9 Soluble guanylate cyclase stimulators

Soluble guanylate cyclase (sGC) is a key enzyme in the NO signalling pathway. sGC catalyses the synthesis of the second messenger cyclic guanosine monophosphate (cGMP), which enhances vasodilation and inhibits smooth muscle proliferation, platelet aggregation, leukocyte recruitment, and vascular remodelling through a number of downstream mechanisms. Deficiency in cGMP causes impaired endothelium-dependent vasomotor regulation in the epicardial and microvascular domain [90]. Currently, 2 sGC-stimulators are available, vericiguat an riociguat and both have been shown to directly stimulate sGC as well as increase sGC sensitivity to endogenous NO and thus enhance the CGMP pathway [91]. sGC are approved in the treatment of pulmonary arterial hypertension. The use of sGC to treat patients with vasomotor disorders may be promising, although the mechanism of action needs to be evaluated. This is currently investigated within the ViVA-trial (EudraCT: 2022–004325-50).

### 4.10 Fasudil

Rho-kinase reduces myosin phosphatase activity by phosphorylating the myosin-binding subunit of the enzyme and thereby augments vascular smooth muscle contraction at a given calcium concentration, which is known as ‘calcium sensitization’ [92]. Increased activity of the Rho-kinase–mediated pathway in vascular smooth muscle cells causes hypercontraction and has been implicated as playing a pathogenetic role in divergent cardiovascular diseases such as coronary artery spasm [93], [94].

Fasudil is a potent and selective inhibitor of Rho-kinase that reduces smooth muscle cell hypercontractility and does not affect heart rate or blood pressure. Although, large randomized studies that evaluate the effect of fasudil in abnormal vasoconstriction are lacking, some smaller studies have indicated that fasudil effectively prevents Ach-induced vasoconstriction in both the epicardial vessels and coronary microvasculature [95], [96], [97].

## 5 Supplementals

### 5.1 Hormone therapy

Several small studies have evaluated the effect of oestrogen therapy in ANOCA patient (angina pectoris, a positive exercise test for myocardial ischemia, and angiographically normal coronary arteries) however results are conflicting. For example, in the multicenter, randomized, placebo-controlled WISE study, 35 women were randomized to receive either 1 mg norethindrone/10 microg ethinyl estradiol or placebo for 12 weeks [98]. Chest pain was significantly less frequent in the treatment group compared with the placebo group (P = 0.02) but no improvement was observed in inducible myocardial ischemia measured by cardiac magnetic resonance spectroscopy nor endothelial dysfunction as assessed by brachial artery reactivity.

### 5.2 Xanthine derivates

Xanthine derivatives are agents that resemble natural occurring xanthines such as caffeine, theobromine and methylxanthines. Mediated by their effects on different tissue phosphodiesterases, xanthines also have other activities including inhibition of platelet function and arterial vasodilation. Theophylline, pentoxifylline, caffeine are xanthine derivatives. Aminophylline is a drug combination of theophylline and ethylenediamine and although not completely understood the mechanisms of actions is mainly via the release of theophylline.

A small study including 8 patients with abnormal vasodilator capacity showed that aminophylline infusion exerted a beneficial effect on exercise-induced chest pain, ischemia-like ECG changes, and increased exercise tolerance compared to placebo infusion [99]. It was hypothesized that aminophylline prevented myocardial flow misdistribution elicited by inappropriate adenosine release during effort in the presence of increased coronary resistance at the level of small intramural coronary arteries. Importantly, as an antidote of dipyridamole-induced vasodilation, high-dose aminophylline (80–240 mg intravenously over 1–3 min) can be used for rapid withdrawal of vasodilation via intravenously. In a small study population high-dose aminophylline was shown to provoke ST-segment elevation accompanied by obvious asynergy detected by echocardiography, in the same electrocardiographic leads showing spontaneous or ergonovine-induced ST-segment elevation [100].

### 5.3 Magnesium

Magnesium is the second in abundance intracellular ion. Magnesium may have a blocking effect on calcium channels and may prevent the contraction of vascular smooth muscle. Magnesium deficiency causes endothelial dysfunction, and hypecoagulopathy. Further depletion seems to be associated with hyperreactivity of coronary arteries to vasoconstrictive stimuli (neurohormonal, electrolytic), whereas magnesium levels normalization plays a role in protection against angina and peripheral vasoconstriction [101]. Magnesium deficiency has been considered as a possible factor contributing to the genesis of coronary vasoconstriction in several reports [102], [103], [104], [105].

As a mechanistic study, the effect of intravenous magnesium sulphate was evaluated in 22 with abnormal epicardial vasoconstriction induced by Ach infusion [106]. After spontaneous relief of the CAS, Magnesium sulphate (0.27 mmol/kg body weight) was infused IV over 20 min in 14 patients and isotonic glucose was infused in 8 patients as control subjects. Treatment with magnesium sulphate caused coronary artery dilatation in both the spastic and non-spastic segments, reduced the severity of chest pain and ST-segment deviations during coronary spasm. Up to date, there are no studies reporting the efficacy of oral treatment with magnesium suppletionin ANOCA patients.

### 5.4 Omega-3 fatty acid

Previous studies have shown that omega-3 fatty acid therapy improves endothelial function in heart transplant recipients and patients with hypercholesterolemia [107], [108]. Moreover, it was shown that omega-3 fatty acid has anti-inflammatory effects and inhibits oxidative stress [109]. In this manner, it has been suggested that omega-3 fatty acid improves endothelial function in the coronary microvasculature. However, robust clinical data is lacking.

### 5.5 L-Arganine

_L_-Arginine is an amino acid produced by the body to help build proteins and is a precursor of NO. Both in vitro and in vivo studies have demonstrated that _L_-arginine supplementation can augment vascular dilation under certain conditions and can improve endothelial function [110]. Most protein-rich foods such as fish, red meat, poultry, soy, whole grains, beans and dairy products are a source of _L_-arginine.

## 6 Other potential therapeutics

### 6.1 Imipramine

Imipramine is a tricyclic antidepressant and affects numerous neurotransmitter systems known to be involved in the aetiology of depression, anxiety, attention-deficit hyperactivity disorder, enuresis and numerous other mental and physical conditions. Imipramine increases levels of serotonin and norepinephrine and blocks certain, adrenergic, histamine, and cholinergic receptors. Moreover, imipramine is similar in structure to some muscle relaxants, and has a significant analgesic effect and, thus, is very useful in some pain conditions. Several small studies have indicated the potential pain reducing effects in ANOCA patients, however a high incidence (75–83 %) of side effects was also reported and no significant improvement of quality of life was established [111], [112].

### 6.2 Pioglitazone

Pioglitazone, a thiazolidinedione and peroxisome proliferator-activated receptor γ (PPAR-γ) agonist, is an anti-diabetic drug to treat type 2 diabetes and works by improving sensitivity of tissues to insulin [113]. PPAR-γ is expressed in a variety of cell types, including vascular smooth muscle cells, endothelial cells, and cardiomyocyte and also have pleiotropic effects. PPAR-γ agonists reduce the activation and inflammation of endothelial cells and endothelial dysfunction. Moreover, PPAR-γ agonists suppress the expression and secretion of ET-1 and enhance NO production providing vasorelaxation. Crosstalk exists between PPAR-γ and NO. An imbalance between endothelium-derived NO and the ET-1 contributes to endothelial dysfunction [114]. The evidence of pioglitazone in ANOCA patients is limited but promising. However, long term use may cause serious side effects including an increased risk for bladder cancer which lead to withdrawal of pioglitazone in several countries. Therefore, the widespread use of pioglitazone is not validated, and long term is not recommended.

### 6.3 Trimetazidine

Trimetazidine is an anti-ischemic metabolic agent of the fatty acid oxidation inhibitor class, and it improves myocardial glucose utilization through inhibition of fatty acid metabolism. In patients with obstructive CAD, studies have shown an increase in CFR and a significant decrease in the frequency of angina attacks. In ANOCA patients, although limited to a small study of 16 patients, Trimetazidine did not exert any significant effect [115].

## 7 Discussion

**Fig. 2:**
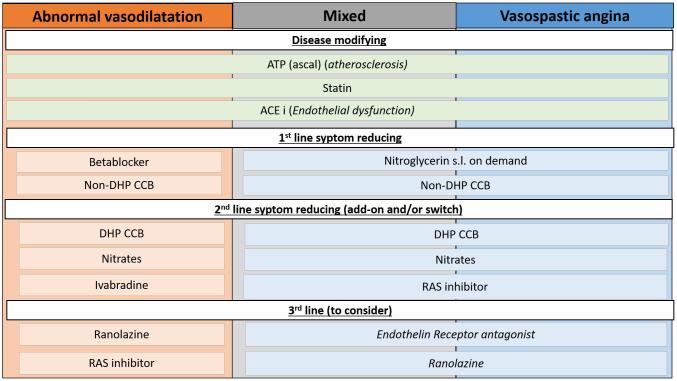
Title: Overview of different pharmacotherapeutic options based on the different vasomotor disorders. Legend: APT (Anti Platelet Therapy); ACE (Angiotensin-converting enzyme); CCB (Calcium Channel Blocker); RAS (Renin Angiotensin System).

Treatment of patients with vasomotor disorder often remain empiric and should be adapted to the underlying pathophysiological mechanism as much as possible. Given the substantial number of patients affected by vasomotor disorders, randomized studies for the evaluation of optimal treatment strategies are needed. In this review, we aimed to provide a general overview of the available literature regarding the pharmacotherapeutic options for the treatment of vasomotor disorders. Depending on the specific vasomotor disorder, we suggest a different pharmacological treatment algorithm (Fig. 2**).** It should be recommended to treat patients with the maximally tolerated dose off the pharmacological agent before the next step is taken. However, challenges remain as due to side effects or clinical signs (hypotension, bradycardia), choices between different pharmacotherapy must be made. Moreover, in our experience, the treatment of patients with a vasomotor disorder is often a road of trial and error. The pharmacological treatment algorithm is currently tested in the prospective randomized ILIAS-ANOCA trial (ICTRP Search Portal (who.int).

### 7.1 General treatment of vasomotor disorders

In general, and regardless of the endotype of vasomotor disorder, statin therapy should be considered in all patients and APT in those with evidence of coronary atherosclerosis. As mentioned before, the underlying pathophysiology and clinical risk factors of vasomotor disorders are generally similar to that of obstructive CAD and the effects seems equally beneficial. Moreover, RAS inhibitors merit general prescription in all ANOCA endotypes to improve endothelial dysfunction.

### 7.2 Treatment of abnormal vasodilatation

In patients with isolated abnormal vasodilation, the aim of treatment is to restore the mismatch between the demand and supply of myocardial oxygen. Beta-blockers are indicated and if there is no improvement of symptoms, non-DHP CCBs (i.e. diltiazem or verapamil) could be considered as an alternative or as an add-on. If the patient remains symptomatic either the beta-blocker can be combined with amlodipine or in case a non-DHP CCB was started and side effects occur the CCB may be switched to amlodipine or barnidipine. The next step would be to add or switch to ivabradine. Although these patients appear to respond less effectively to the administration of sublingual or oral nitrates, nicorandil can be added when the patient still experiences angina. Next would be to add ranolazine and, finally, the RAS inhibitor can be switched to sacubitril/valsartan. It is important to note that in patients with abnormal vasodilatory capacity with predominantly structural properties, beta-blockers and RAS inhibitors might be preferred whereas in the functional endotype, CCB and nitrates may be preferred as first step.

### 7.3 Abnormal vasoconstriction

In all patients with objective abnormal vasoconstriction and in addition to the aforementioned general disease-modifying therapy, nitroglycerin s.L. on demand and a non-DHP CCB should be prescribed. When side effects occur related to non-DHP CCB it may be switched to amlodipine or barnidipine. The second step is to add long-acting nitrates (isosorbide mononitrate) and if insufficient or not tolerated, molsidomine or nicorandil can be started. The next step is to add a RAS inhibitor and if symptoms remain an ERA can be added. Finally, ranolazine can be added. Importantly, the use of beta-adrenergic receptor blockers that do not carry alpha_1_-adrenergic antagonist activity (i.e. all beta-adrenergic receptor blockers except labetalol and carvedilol) should be avoided as they can exacerbate coronary vasospasm.

All other pharmacological agents described in the previous paragraph may be tested in patients with vasomotor disorders.

## 8 Conclusion

Treatment of vasomotor disorders can be very challenging. This document provides the treating clinician/interventional cardiologist an overview of pharmacological agents for vasomotor disorder and a treatment algorithm based on the existing evidence and the best available current practice. Future ongoing research is required to address a number of unanswered questions in the management of these patients.

## Declaration of Competing Interest

The authors declare the following financial interests/personal relationships which may be considered as potential competing interests: [TVH and JJP report consulting fees for Philips. MB received an institutional research grant from Janssen-Cilag B.V. to conduct the VERA-trial. TVH received an institutional research grant from Bayer AG to conduct the ViVA-trial. The remaining authors have nothing to disclose.

].
