# Supplementary material for: Pharmacotherapy in patients with vasomotor disorders
Source: Int J Cardiol Heart Vasc. 2023 Sep 8;48:101267. doi: 10.1016/j.ijcha.2023.101267 (PMC10505589; doi:10.1016/j.ijcha.2023.101267)
Supplement: Supplementary data 1 [file mmc1.docx]

| **Table 1. Overview of evidence regarding therapeutic agents in the treatment of vasomotor disorders** | | | | | | | | | | |
| --- | --- | --- | --- | --- | --- | --- | --- | --- | --- | --- |
| **Drug Class** | **First author** | **Design** | **Patients** | **Year** | **Type of CAD** | **Agents** | **Control** | **Main outcomes** | **Follow-up** | **Side effects** |
| Antiplatelet therapy | *Kim et al.* | Retrospective | N = 424 | 2013 | Abnormal vasoconstriction  (Ergonovine) | Aspirin (<100 mg) at discharge | No-aspirin at discharge | Diffuse vasospasm more frequently in patients without aspirin.  No difference in hospital readmission for angina. | 1 year | NR |
|  | *Ishii et al.* | Retrospective | N=640 | 2016 | Abnormal vasoconstriction (Ach) | Aspirin (<100 mg) | Propensity matched no-aspirin | Use of aspirin was not an independent predictor of MACE. | 5-year | NR |
|  | *Mori et al.* | Retrospective | N=1492 | 2020 | Abnormal vasoconstriction (Ach, Ergonovine or objective angina with ECG changes and NOCAD) | Aspirin (<100 mg) or P2Y12 inhibitor | Propensity matched no-antiplatelet | Incidence of MACE comparable between group with aspirin and group without aspirin. | 32 months | NR |
|  | *Cho et al.* | Prospective | N=1838 | 2019 | Abnormal vasoconstriction (Ergonovine) | Aspirin;  Clopidogrel,;  DAPT | Non-antiplatelet | Composite outcome (death from any cause, ACS and symptomatic arrhythmia) more common in DAPT group.  Aspirin or Clopidogrel alone similar event rates to no-aspirin group. | 3 year | NR |
| Statins | Tani, et al. | Prospective pilot study | N = 27 | 2008 | Abnormal vasoconstriction (Ach) | Pravastatin (10-20 mg daily) in addition to CCB | None | 48% reduction in Ach induced vasoconstriction  Improvement in serum lipid levels and decrease in serum MDA-LDL level | 6 months | NR |
|  | Yasue, et al. | Prospective, randomized, open-label | N = 64 | 2008 | Abnormal vasoconstriction (Ach) | Fluvastatin (30 mg o.d.) + CCB (Diltiazem ER 100-200 mg or nifedipine ER 20-40 mg) | CCB alone | Significant suppression of Ach induced abnormal vasoconstriction in fluvastatin group (52% vs 21%, p = 0.0231) | 6 months | NR |
|  | ishii, et al. | Retrospective | N = 640 | 2016 | Abnormal vasoconstriction (Ach) | Statin (any) | Propensity matched no-statin | Statin therapy was an independent negative predictor of composite of cardiac death, nonfatal MI, unstable angina. | 5-year | NR |
|  | Oh, et al. | Retrospective | N = 562 | 2016 | Abnormal vasoconstriction (Ach) | Statin (any) | Propensity matched no-statin | No difference between statin and no statin group in composite of cardiovascular death, myocardial infarction and any revascularization and readmission due to chest pain. | 4.5 years | NR |
|  | Park et al. | Retrospective | N = 4099 | 2019 | Abnormal vasoconstriction (Ach) | Statin (any) | Propensity matched no-statin | No difference in occurrence of composite of cardiac arrest and acute myocardial infarction (5.2% vs 5.4%, p = 0.976) | 3.8 years | NR |
|  | Seo et al. | Retropsective | N = 1713 | 2020 | Abnormal vasoconstriction (Ach) | Statin (any) | Propensity matched no-statin | No difference in occurrence of composite outcome of cardiac death, acute coronary syndrome, and new-onset-life-threatening arrhythmia | 3 years | NR |
| Renin-Angiotensin System Inhibitors | Pizzi et al. | Prospective, randomized, placebo controlled | N = 45 | 2004 | Abnormal vasodilation (Syndrome X; no abnormal vasoconstriction) | Ramipril (10 mg o.d.) + atorvastatin (40 mg o.d.) | Placebo | Improvement of exercise duration (by 23.46%) and SAQ (by 64.1%) compared to placebo | 6 months | NR |
|  | Nalbantgil et al. | Prospective, randomized, placebo controlled | N = 18 | 1998 | Abnormal vasodilatation  (Syndrome X; no abnormal vasoconstriction) | Cilazapril (2.5 mg t.d.) | Placebo | Significant decrease in magnitude of ST-segment depression and prolongation of total exercise time with Cilazapril therapy. | 6 weeks | NR |
|  | Chen et al. | Propsective, randomized, placebo controlled | N = 20 | 2002 | Abnormal vasodilatation  (Syndrome X; no abnormal vasoconstriction) | Enalapril (5 mg t.d.) | Placebo | Exercise duration and CFR significantly improved with enalapril, but not with placebo  Enalapril reduced von Willebrand factor and ADMA levels, and increased NOx levels | 8 weeks | NR |
|  | Pauly et al. | Prospective, randomized, placebo controlled | N = 78 (women) | 2011 | Abnormal vasodilatation (CFR < 3.0) | Quinapril | Placebo | CFR improved more with Quinapril than with placebo, improvement was associated with reduction in angina | 16 weeks | NR |
|  | Choi et al. | Retropsective | N = 3349 | 2016 | Abnormal vasoconstriction (Ach) | RAS inhibitor (any) | Propensity matched no RAS inhibitor | RAS group showed a lower incidence of recurrent angina, all-cause mortality, and MACE (Death, MI or de novo PCI ) | 5 years | NR |
|  | Russel et al. | Prospective, randomized, placebo | N = 28 | 2007 | Abnormal vasodilatation (Syndrome X) | Irbesartan (150 mg o.d.) | Placebo | Irbesartan produced no significant subjective or objective improvements in ANOCA patietns (difference in angina attacks, NTG consumption or exercise duration) | 6 weeks | NR |
| Calcium Channel Blockers | Muller et al. | Case report | N = 1 | 1978 | Abnormal vasoconstriction (Prinzmetal angina) | Nifedipine | None | Relief of symptoms | 1 month | NR |
|  | Antman et al. | Prospective | N = 127 | 1978 | Abnormal vasoconstriction | Nifedipine (40 – 160 mg daily) | None | Decreased frequency of angina attacks, marked reduction of NTG s.l. use | NR | 5 patient required termination of the drug due intolerable side effects |
|  | Battye et al. | Retrospective | N = 21 | 1979 | Abnormal vasoconstriction (Prinzmetal angina) | Nifedipine (30 – 120 mg dialy) | None | Reduction in the number of anginal attacks per day (p<0.001) with nifedipine. | NR | Recorded side-effects were of minimal severity and in no case caused nifedipine therapy to be discontinued. |
|  | Nishigaki et al. | Meta-analysis | N = 1997 | 2010 | Abnormal vasoconstriction (Ach or Ergonovine) | Benidipine  Amlodipine  Nifedipine  Diltiazem | None | Occurrence of MACE significantly lower in those treated with benidipine compared to the other groups | Median 4.4 years | NR |
|  | Schick et al. | Prospective, randomized, withdrawal | N = 38 | 1982 | Abnormal vasoconstriction (Prinzmetal Angina or Ach) | Nifedipine | Placebo | Significant increase in angina attacks in placebo group compared to Nifedipine group (study prematurely stopped) | 6 week | Mild side effects reported, no discontinuation needed |
|  | Chahine et al. | Prospective, randomized, placebo controlled | N= 52 | 1993 | Abnormal vasoconstriction (Prinzmetal angina or Ergonovine) | Amlodipine (10 mg daily) | Placebo | Anginal episodes and NTG intake decreased significantly in amlodipine group compared to placebo.  Decrease in anginal attacks after 1 year | 4 week and 1 year (N=29) | Mild side effects reported, no discontinuation needed |
|  | Johnson et al. | Prospective, randomized, placebo controlled | N = 16 | 1981 | Abnormal vasoconstriction (Variant angina) | Verapamil | Placebo | Frequency of angina and NTG use decreased significantly with verapamil compared to placebo.  Lower incidence of hospitalizations and transient ST-segment deviation with verapamil | 9 months | No adverse effects forcing a reduction in dosage or discontinuation |
|  | Jansen et al. | Prospective, randomized, placebo controlled | N = 126 | 2022 | Abnormal vasodilation, abnormal vasoconstriction or mixed (ANOCA) | Diltiazem | Placebo | No difference in CFT outcome and no difference in SAQ or RAND-36 questionnaire  Diltiazem progress disease form epicardial to microvascular vasoconstriction | 6 weeks | NR |
|  | Prinzmetal et al. | Propsective, randomized, placebo controlled | N = 12 | 1959 | Abnormal vasoconstriction (Prinzmetal angina) | Diltiazem (120 – 240 mg/day) | Placebo | Significant decrease in angina frequency by diltiazem | 18 months | NR |
|  | Pesola eta l. | Prospective, randomized, placebo controlled | N = 10 | 1987 | Abnormal vasoconstriction (variant angina) | Diltiazem (60 mg) | Placebo | Signficant decrease in ischemic episodes on continuous Holter monitoring by Diltiazem | 72 hours | NR |
| Nitrates | Conti et al. | Prospective, cross-over | N = 19 | 1985 | Abnormal vasoconstriction (variant angina) | ISDN (40-120 mg)  Nifedipine (40 – 120 mg) | None | ISDN and Nifedipine equally effective in controlling angina | 7 weeks | Mild side-effects reported |
|  | Russo et al. | Cross-sectional | N = 29 | 2013 | Abnormal vasodilatation (INOCA) | ISDN (5 mg s.l.) | None | Short-acting ISDN did not improve exercise stress test outcomes | N/A | NR |
|  | Wu et al. | Prospective, randomized, placebo controlled | N = 20 | 2015 | Abnormal vasodilatation (INOCA; no abnormal vasoconstriction) | Isosorbide-5-mononitrate (30 mg o.d.) in addition to beta-blocker or CCB | Placebo | Small significant difference in SAQ and European QoL score in those patients that completed the study.  No difference in exercise stress testing | 4 week | 45% of patients did not complete the study due to side effects |
|  | Chen et al. | Prospective, randomized, placebo controlled, cross-over | N = 13 | 1997 | Abnormal vasodilatation (INOCA; no abnormal vasoconstriction) | Nicorandil (5 mg, t.d.) | Placebo | Nicorandil improved exercise duration and time to 1-mm ST depression during exercise testing. | 2 weeks | NR |
|  | Jia et al. | Meta-analysis | N = 2323 | 2020 | Abnormal vasodilatation (INOCA; no abnormal vasoconstriction) | Nicorandil | N/A | Improvement of angina symptoms, resting ECG, treadmill test results and endothelial function by Nicorandil. | NR | No serious adverse drug events |
| Beta-blocker | Robertson et al. | Prospective, placebo controlled crossove | N = 6 | 1982 | Abnormal vasoconstriction | Propranolol (40 & 160 mg) | Placebo | Duration of angina attack was significantly prolonged with propranolol, frequency was not | 3 days | NR |
|  | Antman et al. | Prospective | N = 127 | 1980 | Abnormal vasoconstriction | Propranolol | NR | Small group found propranolol partially effective for alleviating symptoms (19%), majority of patients (69%) found propranolol ineffective and some patietns (9.5%) experienced increased frequency of angina. | NR | NR |
|  | Kook et al. | Prospective, randomized | N = 51 | 2020 | Abnormal vasoconstriction (Ach) | Nebivolol only;  Diltiazem only;  Combination | None | All groups showed significant improvement in percent changes of coronary vasoconstriction.  Improvement was greatest in Diltiazem only group.  No difference in SAQ score between the three groups. | 12 weeks |  |
| α1-adrenergic receptor antagonist |  |  |  |  |  |  |  |  |  |  |
|  | Yasue et al. | Observational | N = 26 | 1978 | Abnormal vasoconstriction (Prinzmetal) | Propranolol;  Diltiazem;  Dipyridamole;  Atropine;  Phenoxybenzamin | None | Propranolol aggravated sympotms, Diltiazem supressed symptoms completely, Dipyridamole was ineffective, atropine and phenoxybenzamine suppressed symptoms | NR | NR |
|  | Tzivoni et al. | Observational | N = 6 | 1983 | Abnormal vasoconstriction (Prinzmetal) | Prazosin (8 – 30 mg daily) in addition to low-dose nitrates or nifedipine | None | Addition of Prazosin abolished anginal attacks in 4 patients an markedly reduced frequency and intensity in 1. | 4-6 months | 1 patient stopped because of hypotension |
|  | Winniford et al. | Prospective, randomized, placebo controlled | N = 6 (men) | 1983 | Abnormal vasoconstriction (Prinzmetal) | Prazosin (9 – 15 mg daily) | Placebo | Prazosin did not change the weekly number of chest pain episodes, NTG use, or transient ST deviations | 4,5 months | 4 patients reported side effects of hypotension |
|  | Rosen et al. | Prospective, randomized, placebo controlled | N = 16 | 1999 | Abnormal vasodilatation (Syndrome X) | Doxazosin ( 1 – 4 mg) | Placebo | Doxazosin decreased systolic blood pressure and increased basal heart rate.  No difference between time to angina, exercise duration and time to ST-segment deviation | 10 weeks | NR |
| Ivabradine | Vilano et al. | Prospective, randomized, placebo controlled | N = 46 | 2013 | Abnormal vasodilatation (CFR <2.5) | Ivabradine;  Ranolazine; | Placebo | Ivabradine significantly improved SAQ and EuroQoL compared to placebo |  |  |
| Ranolazine | n/a |  | n/a | n/a | n/a | n/a | n/a | n/a | n/a | n/a |
| Cilostazol | Watanabe et al. |  | N = 30 | 2003 | Abnormal vasoconstriction | Cilostazol | No treatment or aspirin | Cilostazol increased CFR, ad diameter changes by L-NMMA compared to the other groups. |  |  |
|  | Shin et al. |  | N = 49 | 2014 | Abnormal vasoconstriction | Cilostazol in addition to amlodipine (5 mg daily) | Amlodipine + placebo | Reduction of weekly incidence of chest pain was significantly greater in the group with cilostazol |  |  |
| Fasudil | Masumoto et al. |  | N = 20 | 2002 | Abnormal vasoconstriction | Intracoronary fasudil | Intracoronary saline | Fasudil prevented the occurrence of chest pain and ischemic ECG changes in all treated patients |  |  |
|  | Otsuka et al. |  | N = 26 | 2008 | Abnormal vasoconstriction | Intravenous fasudil | Intracoronary NTG, intravenous saline | Fasudil and not saline further dilated the diameter observed at the location of vasoconstriction in addition to intracoronary NTG |  |  |
|  | Mohri et al. |  | N = 18 | 2003 | Abnormal vasoconstriction | Fasudil intracoronary | Saline | Fasudil ameliorated myocardial ischemia |  |  |
| Hormone therapy | Ademson et al. |  | N = 19 (women) | 2001 | ANOCA | Esterified estrogens | Placebo | Estrogens had beneficial effect on emotional well-being, not on exercise parameters or chest pain |  |  |
|  | Albertsson et al. |  | N = 15 (post menopauzal women) | 1996 | ANOCA | Transdermal estradiol | Placebo | Estrogen increased time to angina, time to ST depression, total exercise time and working capacity. |  |  |
|  | Rosano et al. |  | N = 25 (post-menopauzal women | 1996 | ANOCA | 17-beta-estradiol transdermal | Placebo | Reduction in anginal episodes, but nog difference in exercise duration |  |  |
|  | Merz et al. |  | N = 35 (women) | 2010 | INOCA | Estradiol | Placebo | Chest pain was significantly less frequent in Estradiol group, no improvement in inducible myocardial ischemia | 12 weeks |  |
| Xanthine derivates | Emdin et al. |  | N = 8 | 1989 | Abnormal vasoconstriction | Aminopohylline | Placebo | Aminophylline infusion exerted a beneficial effect on exercise-induced chest pain, ischemia-like ECG changes, and increased effort tolerance |  |  |
|  | Picano et al. |  | N = | 1988 | Abnormal vasoconstriction | Aminophylline |  | High-dose aminophylline was shown to provoke ST-segment elevation |  |  |
| Magnesium | Teragawa et al. |  | N = 22 | 2000 | Abnormal vasoconstriction | Magnesium sulfate intra coronary | Isotonic glucose intracoronary | Magnesium caused coronary artery dilatation, reduced severity of chest pain and ST-segment deviations during provocation |  |  |
| Omega-3 fatty acid | Bozcali et al. |  | N = 18 | 2013 | ANOCA | Omega-3 fatty acids | Placebo | Omega-3 showed a significant increase in flow-mediated dilatation and NTG mediated dilatation and a significant decrease in plasma malondialdehyde levels. |  |  |
| l-Arganine | Egashira et al. |  | N = 8 | 1996 | ANOCA | l-arginine intracoronary | Non-ANOCA controls | l-arginine significantly augmented CBF responses to Ach in ANOCA patients, but not in controls |  |  |
|  | Lerman et al. |  | N = 26 | 1998 | ANOCA | l-Arginine (3g t.i.d.) | Placebo | L-Arginine increased CBF and decreased plasma endothelin in response to Ach. Patient symptoms improved by l-Arginine | 6 months |  |
| Imipramine | Cannon et al. |  | N = 40 | 1994 | ANOCA | Imipramine (50 mg) | Placebo | Imipramine significantly reduced chest pain | 3 week | 75% reported side effects of imipramine |
|  | Cox et al. |  | N = 18 (female) | 1998 | ANOCA | Imipramine (50 mg daily) | Placebo | Imipramine significantly reduced anginal symptoms | 5 week | 83% reported side effects, 3 patients withdrawn from treatment |
| Pioglitazone | Morita et al. |  | N = 73 | 2014 | Abnormal vasoconstriction | Pioglitazone (15 – 30 mg daily) added on CCBS | CCB alone | Abnormal vasoconstriction was supressed in 50% in the pioglitazone group compared to 21.6% in the control group | 6 month |  |
| Trimetazidine | Leonardo et al. |  | N = 16 | 1999 | ANOCA | Trimetazidine (20 mg t.i.d.) | Atenolol (100 mg) or placebo | Trimetazidine did not exert any significant effect compared to the other groups | 2 weeks |  |
